# Harmine Derivative H‐2‐104 Alleviates *Echinococcus granulosus*‐Induced Pulmonary Fibrosis in Mice by Inhibiting Fibroblast Activation and Inducing DNA Damage

**DOI:** 10.1155/jimr/8865338

**Published:** 2026-09-24

**Authors:** Yuehong Gong, Yating Cui, Miechi Pan, Haibo Zhang, Yisikandier Abudusaimaiti, Guohua Tang, Hang Ren, Lijie Fu, Jianhua Yang, Junping Hu

**Affiliations:** ^1^ Department of Pharmacognosy, Xinjiang Medical University, First Affiliated Hospital of Xinjiang Medical University, Urumqi 830011, Xinjiang, China, xjmu.edu.cn; ^2^ Department of Pharmacy, Xinjiang Key Laboratory of Clinical Drug Research, First Affiliated Hospital of Xinjiang Medical University, Urumqi 830011, Xinjiang, China, xjmu.edu.cn; ^3^ College of Pharmacy, Xinjiang Medical University, Urumqi 830017, Xinjiang, China, xjmu.edu.cn; ^4^ School of Clinical Medicine, Xinjiang Medical University, Urumqi 830017, Xinjiang, China, xjmu.edu.cn

**Keywords:** DNA damage, *Echinococcus granulosus*, fibroblast activation, H-2-104, pulmonary fibrosis, TGF-β1

## Abstract

**Background:**

Pulmonary fibrosis (PF) secondary to *Echinococcus granulosus* infection is a severe complication with limited therapeutic options. Harmine (HM) exhibits antifibrotic potential but is restricted by significant neurotoxicity. This study aimed to evaluate the therapeutic efficacy and underlying mechanism of HM derivative H‐2‐104 in a clinically relevant *E. granulosus*‐induced PF mouse model, and to clarify its advantages over parent compound HM.

**Methods:**

A mouse model of *E. granulosus*‐induced PF was established, and treated with H‐2‐104 (25 or 50 mg/kg) or nintedanib for 21 days. Lung function, histopathology, hydroxyproline (HYP), inflammatory cytokines, and fibrosis‐related protein expression were assessed. In vitro, human embryonic lung fibroblasts (MRC‐5) were activated with TGF‐β1 and treated with H‐2‐104. Cell viability, migration, apoptosis, differentiation, DNA damage, and related gene expression were evaluated.

**Results:**

H‐2‐104 significantly improved lung function, alleviated pathological damage, and collagen deposition, decreased HYP, TGF‐β1, TNF‐α, IL‐6, and IL‐1β, elevated IL‐10, and downregulated α‐smooth muscle actin (α‐SMA), collagen I, and fibronectin. Importantly, H‐2‐104 showed no obvious neurotoxicity and exhibited better safety than HM. In vitro, H‐2‐104 inhibited TGF‐β1‐induced MRC‐5 cell viability (IC_50_ = 13.49 µg/mL), migration, and fibroblast‐to‐myofibroblast differentiation, while promoting apoptosis along with increased DNA damage in a dose‐dependent manner. Additionally, H‐2‐104 downregulated the expression of fibrosis‐related genes and upregulated DNA damage‐related genes (*H2AX*, *ATR*, and *RAD51*) in TGF‐β1‐induced MRC‐5 cells.

**Conclusion:**

This study establishes H‐2‐104 as a promising antifibrotic candidate for *E. granulosus*‐induced PF, using a clinically meaningful parasitic infection‐associated fibrosis model. H‐2‐104 retains strong antifibrotic efficacy while overcoming the neurotoxicity limitation of HM. Mechanistically, the activation of DNA damage response is correlated with the antifibrotic activities of H‐2‐104, providing a new perspective for targeting fibroblast activation in PF therapy.

## 1. Introduction

Echinococcosis is a neglected zoonotic parasitic disease caused by the larval stages of *Echinococcus* species, posing a severe threat to global public health and animal husbandry, particularly in the pastoral and semi‐pastoral regions of Central Asia, Western China, and South America [1, 2]. Among the various clinical manifestations of echinococcosis, pulmonary echinococcosis (PE) accounts for approximately 20%–30% of human cases, second only to hepatic echinococcosis [3, 4]. *Echinococcus granulosus* (*E. granulosus*), the primary etiological agent of cystic echinococcosis (CE), can migrate to the lungs via the systemic circulation, where the developing hydatid cysts trigger a series of local inflammatory and fibrotic responses [5]. Pulmonary fibrosis (PF) secondary to *E. granulosus* infection has emerged as a major complication that impairs lung function, increases the risk of respiratory failure, and significantly reduces patients’ quality of life [6, 7].

However, *E. granulosus* infection can cause severe secondary PF, which seriously impairs lung function and increases mortality. Currently, effective clinical interventions are still limited. Notably, the crosstalk between fibroblast activation and the DNA damage response in *E. granulosus*‐induced PF remains a largely underexplored mechanistic gap. This missing key link restricts the development of precise therapeutic targets, emphasizing the urgent need to explore this pathway for novel antifibrotic drug development.

PF is characterized by abnormal fibroblast activation, tissue remodeling, and excessive extracellular matrix (ECM) deposition [8, 9]. The excessive accumulation of ECM components, such as collagen I and fibronectin, disrupts the normal alveolar structure [10]. Fibroblast activation is a central event in the pathogenesis of PF, transforming quiescent fibroblasts into myofibroblasts, highly proliferative cells that secrete large amounts of ECM [11]. Myofibroblasts are identified by the expression of α‐smooth muscle actin (α‐SMA), and their persistent activation is considered a hallmark of progressive fibrosis [12]. In *E. granulosus* infection, the cyst fluid, protoscoleces, and parasitic antigens can induce the release of pro‐inflammatory cytokines (e.g., TNF‐α and IL‐6) from local immune cells and epithelial cells [13, 14]. Among these, TGF‐β1 is a key mediator that activates the Smad signaling pathway, thereby promoting fibroblast proliferation, differentiation into myofibroblasts, and ECM synthesis [15, 16]. Therefore, inhibiting fibroblast activation, the downstream TGF‐β1‐mediated signaling cascade, and the associated DNA damage response have become a promising therapeutic direction for *E. granulosus*‐induced PF.

Currently, only two antifibrotic drugs, nintedanib and pirfenidone, are approved to slow PF progression in humans, and both are associated with considerable adverse effects. Lung transplantation is limited by surgical risks, rejection, and donor shortage. Thus, there is a strong clinical demand for safer and more effective novel therapeutic agents. Harmine (HM), a β‐carboline alkaloid isolated from the seeds of *Peganum harmala* L., has garnered increasing attention due to its diverse biological activities, including antiparasitic, anti‐inflammatory, and antitumor effects [17]. Notably, β‐carboline alkaloids have been reported to exhibit promising antifibrotic properties, as well as HM could alleviate weight loss and improve lung function in a mouse model of PF by regulating DDR‐related genes and activating the TP53‐Gadd45α pathway, thereby suppressing PF [18]. Another investigation has demonstrated that HM can inhibit COL1A1 expression by targeting DYRK1B in hepatic stellate cells, suggesting its potential efficacy in treating liver fibrosis [19]. These findings suggest that HM is a promising antifibrotic agent. However, it has been reported that HM exhibits potent neurotoxicity, which can stimulate the central nervous system and induce adverse reactions, even life‐threatening conditions, in humans and animals, thus limiting its clinical application [20]. To reduce the neurotoxicity of HM, our research team previously modified its chemical structure to obtain the HM derivative H‐2‐104, another member of the β‐carboline alkaloid family. The antifibrotic activity of H‐2‐104 has been documented in prior research [18]. A preliminary study has reported that H‐2‐104 possesses favorable absorption characteristics, high bioavailability, and low cytotoxicity [21]. Furthermore, data have indicated that H‐2‐104 exerts significant inhibitory activity against *E. granulosus*, suggesting that it may represent a promising novel drug for the treatment of CE [22]. Additionally, its anti‐CE effects may be attributed to the regulation of multiple biological pathways, including apoptosis, amino acid metabolism, and glucose metabolism [22]. Nevertheless, the therapeutic role and underlying mechanism of H‐2‐104 against *E. granulosus*‐induced PF remain unclarified. Therefore, in‐depth research into the potential of HM derivatives in improving PF will expand their pharmaceutical applications and provide new directions for the management of this disease.

Therefore, the present study established an *E. granulosus*‐induced PF mouse model and TGF‐β1‐activated MRC‐5 cell model to evaluate the therapeutic effect of HM derivative H‐2‐104 on PF and explore its underlying mechanism related to fibroblast activation and DNA damage response. The results of this study will provide new insights into the development of novel therapeutic agents for *E. granulosus*‐induced PF.

## 2. Materials and Methods

### 2.1. Collection and In Vitro Culture of *E. granulosus*

Fresh sheep livers naturally infected with *E. granulosus* were obtained from Hualing Slaughterhouse in Urumqi, Xinjiang, China. Under sterile conditions, the inner cysts were isolated from the hepatic cysts of sheep livers, and the cyst fluid was aspirated. The inner cysts were then incised and rinsed repeatedly with sterile normal saline. After precipitation, *E. granulosus* were collected, and their morphology and viability were examined to ensure a viability rate of ≥95%. The collected *E. granulosus* were digested with 1% pepsin (pH 2.0, Sigma, USA), filtered to remove impurities and poorly viable *E. granulosus*, and subsequently washed repeatedly with normal saline supplemented with 2% penicillin/streptomycin (Thermo Fisher Scientific, USA). Thereafter, *E. granulosus* with good viability were transferred to new culture flasks containing complete medium and incubated in a constant‐temperature incubator at 37°C with 5% CO_2_. The medium consisted of RPMI‐1640 (Thermo Fisher Scientific) supplemented with 10% fetal bovine serum (FBS, Thermo Fisher Scientific) and 2% penicillin/streptomycin, and the medium was refreshed every 3 days.

### 2.2. Establishment of a *E. granulosus*‐Induced PF Model in Mice and Grouping

Fifty C57BL/6 mice aged 8‐week‐old and weighing 16–18 g were purchased from Animal Experimental Center of Xinjiang Medical University (Xinjiang, China) and maintained in Animal Experimental Center of Xinjiang Medical University (the production license for experimental animals: SCXK [Xin] 2023‐0001, and the use license for experimental animals: SYXK [Xin] 2023‐0004.). Furthermore, all the mice were fed in a specific pathogen free (SPF) environment with a constant temperature of 22 ± 2°C, relative humidity of 55% ± 5%, and a 12 h light/dark cycle. During the experiments, all the mice had free access to drink and food. This study was reviewed and approved by the Ethics Committee of the Animal Center of Xinjiang Medical University, and all procedures were performed in strict accordance with ethical standards (approval no.: IACUC‐20230321‐54).

After 7 days of acclimatization, all the mice were randomly divided into five groups (*n* = 10 for each group): control, model, nintedanib, 25 mg/kg H‐2‐104, and 50 mg/kg H‐2‐104 groups. Except for the control group, the mice in the other groups were infected by an intraperitoneal injection of *E. granulosus* with a vitality of ≥95%. After 5 months of infection, the mice gained weight, their abdomen swelled, and dissection showed vesicles in their bodies, indicating successful modeling. After successful modeling, the mice in the control, model, nintedanib, 25 mg/kg H‐2‐104 (cat. No. 181022, purity ≥98%, Xinjiang Huashidan Pharmaceutical Co., Ltd, Xinjiang, China), and 50 mg/kg H‐2‐104 groups were administered with the equal volume of 0.5% CMC‐Na solution, the equal volume of 0.5% CMC‐Na solution, 30 mg/kg nintedanib, 25 mg/kg H‐2‐104, and 50 mg/kg H‐2‐104 by gavage for 21 days, respectively. Body weight of the mice was measured every 3 days.

### 2.3. Mouse Lung Function Test and Blood Biochemical Examination

On day 21 of the experiment, all the mice were placed in the plethysmograph chamber of a noninvasive pulmonary function testing system for consecutive 3 days to measure pulmonary function, with all procedures performed in accordance with the operational standards of the EMMSLink WBP system (EMMS company, UK). Pulmonary function parameters of each mouse included respiratory frequency, minute ventilation (MV), expiratory flow at 50% ventilation (EF50), and maximum expiratory flow rate (PEF).

Three days after the consecutive pulmonary function measurements (day 24 of the experiment), mice were fasted for 12 h before the body weight was recorded. Anesthesia was induced by an intraperitoneal injection of sodium pentobarbital, followed by orbital blood collection. The blood samples were centrifuged at 861 × *g* for 15 min at 4°C, and the supernatant (serum) was collected for blood biochemical analysis. The detected parameters contained alanine aminotransferase (ALT), aspartate aminotransferase (AST), urea (UREA), and creatinine (CREA). After orbital blood sampling, the mouse lung tissues, liver tissues, kidney tissues, and brain tissues were harvested and stored in liquid nitrogen for subsequent use.

### 2.4. Histopathologic Examination of Lung Tissues in Mice

Lung, liver, kidney, and brain tissues were selected from three mice per group and were perfused with 4% paraformaldehyde solution, followed by immersion in paraformaldehyde for further fixation for 72 h. Subsequently, the tissues underwent fixation, dehydration, paraffin embedding, and sectioning into 4‐μm‐thick slices. After that, these tissues were stained with hematoxylin and eosin (HE), and the lung and liver tissue samples were employed for Masson’s trichrome staining. After being mounted, pathological observations were performed under a light microscope.

All histological examinations and semi‐quantitative scoring were performed in a blinded manner by two independent experienced pathologists who were unaware of the animal grouping information. Semi‐quantitative scoring of lung pathological sections was performed using NIS‐Elements 4.6 software. The scoring indices included inflammatory cell infiltration, alveolar septal thickness and fibrotic area, with scores defined as follows: 0 = normal lung tissue; 1 = slight fibrosis with mild alveolar septal thickening; 2 = mild fibrosis with obvious alveolar septal thickening and intact alveolar structure; 3 = moderate fibrosis, damaged alveolar structure and small fibrotic foci; 4 = moderate to severe fibrosis with multiple fused fibrotic foci; 5 = severe fibrosis with extensive lung destruction and residual partial alveolar and bronchial structures; 6 = advanced fibrosis with completely destroyed lung architecture and large unstructured fibrotic regions; 7 = extremely severe fibrosis, with tissue dominated by fibrotic tissue and only a few gaps left; and 8 = complete remodeling of lung architecture with the entire field of view occupied by fibrous tissue. Six lung sections were selected per group, and three random visual fields were analyzed for each section. All scoring data were subjected to statistical analysis. Semi‐quantitative scoring for liver fibrosis was also assessed via Masson’s trichrome staining using the same semi‐quantitative scoring criteria.

### 2.5. Determination of Hydroxyproline (HYP) and Related Inflammatory Cytokines in Lung Tissues

The right upper lung or the liver tissue samples of each mouse (0.08 g) were collected and dissolved in 6 mol/L hydrochloric acid extract for carbonization. After adjusting the pH to 6–8, the contents of HYP (HYP) in lung or liver tissues were measured using a HYP Assay Kit (Nanjing Jiancheng Bioengineering Institute, Nanjing, China) based on the alkaline hydrolysis method in accordance with the manufacturer’s instructions.

Furthermore, the levels of inflammatory cytokines in lung tissues or blood samples, including IL‐10, TGF‐β1, TNF‐α, IL‐6, and IL‐1β, were examined by enzyme linked immunosorbent assay (ELISA) with their corresponding ELISA assay kits (FineTest, Wuhan, China) following the protocols of the manufacturer.

### 2.6. Western Blot

Lung tissues in different groups (0.1 g) were weighed and homogenized in lysis buffer via grinding in liquid nitrogen until a powder was formed. After centrifugation at 1705 × *g* for 5 min, total proteins were extracted from the lung tissues, and protein concentrations were quantified using a BCA protein assay kit (Solarbio, Beijing, China). Subsequently, the isolated protein samples (20 μg) were subjected to sodium dodecyl sulfate–polyacrylamide gel electrophoresis (SDS–PAGE). Following separation, proteins were transferred onto polyvinylidene fluoride (PVDF) membranes, which were then blocked with 5% (*w*/*v*) bovine serum albumin (BSA) at room temperature for 1 h. The membranes were incubated overnight at 4°C with primary antibodies against α‐SMA (1:1000, Proteintech, Wuhan, China), collagen type Ⅰ (collagen Ⅰ, 1:1000, Proteintech), fibronectin (1:1000, Proteintech), and β‐actin (1:2000, Proteintech). After washing, the membranes were incubated with horseradish peroxidase (HRP)‐conjugated secondary antibodies (1:2000, Bioss, Beijing, China) for 1 h at room temperature in the dark. Subsequently, the membranes were washed with tris‐buffered saline with Tween‐20 (TBST) and subjected to chemiluminescence development. The initial exposure time was set at 10 s to 5 min, and adjustments were made based on the results until clear protein bands were obtained. The bands were scanned and saved, and gray value analysis was performed using ImageJ software with β‐actin as an internal reference.

### 2.7. Cell Culture and Cell Viability Assay

Human embryonic lung fibroblast MRC‐5 cells (cat. No. CL‐0161) were purchased from Procell (Wuhan, China) and maintained in MEM medium (Thermo Fisher Scientific) containing 10% FBS and 1% penicillin–streptomycin at 37°C and 5% CO_2_.

Recombinant human transforming growth factor‐β1 (TGF‐β1) was purchased from Wuhan Fine Biotech Co., Ltd (Wuhan, China). Recombinant human TGF‐β1 was used as the fibrotic inducer. Lyophilized TGF‐β1 powder was dissolved in sterile phosphate‐buffered saline (PBS) to prepare a 10 μg/mL stock solution. The stock solution was divided into small aliquots and stored at −80°C. Prior to experiments, the stock solution was diluted with a fresh complete medium to a final working concentration of 5 ng/mL [23].

After routine culture of MRC‐5 cells, logarithmic‐phase cells were seeded into 96‐well plates at a density of 5 × 10^3^ cells/well and divided into eight groups: blank control group (complete medium only), TGF‐β1 group (cells induced with 5 ng/mL TGF‐β1), and TGF‐β1 + H‐2‐104 groups (cells induced with 5 ng/mL TGF‐β1 and then intervened with 0.00, 1.56, 3.13, 6.25, 12.50, 25.00, 50.00, and 100.00 μg/mL H‐2‐104). Each well was administered 100 μL of the corresponding treatment. After 24 h of incubation, 10 μL of cell counting kit‐8 (CCK‐8, Beyotime, Shanghai, China) solution was added to each well, followed by a 4‐h incubation. The optical density (OD) value at 450 nm was measured using a microplate reader (Berthold, Germany), and cell viability and the half‐maximal inhibitory concentration (IC_50_) were calculated. The formula for cell viability was as follows:

 Cell viability%=OD450 of experimental group−OD450 of blank group/OD450 of control group−OD450 of blank group×100%.

Based on the IC_50_ value of H‐2‐104, the concentration corresponding to the IC_50_ and its two adjacent concentrations were selected for gradient intervention. The CCK‐8 assay was repeated to further verify the effect of H‐2‐104 on the viability of TGF‐β1‐induced MRC‐5 cells.

### 2.8. Measurement of Cell Migration, Apoptosis, and Differentiation

The migration, apoptosis, and differentiation of MRC‐5 cells were, respectively, determined using scratch assay, flow cytometry and immunofluorescence (IF). For cell migration, logarithmic‐phase MRC‐5 cells were seeded into 12‐well plates at a density of 1 × 10^4^ cells/mL and cultured for 24 h. When the cell confluence reached 80% or higher, vertical scratches were made using a 100 μL pipette tip. After washing three times with PBS, 2.5 mL of serum‐free medium was added to each well. The cells were then treated with TGF‐β1 (5 ng/mL) and H‐2‐104 at final concentrations of 7, 14, and 28 μg/mL. The migration status of cells in each group was photographed under a microscope immediately after scratching (0 h) and at 24 h post‐scratching. ImageJ software was used to quantify the scratch area and calculate the cell migration rate. The formula for the cell migration rate was as follows:

Cell migration rate %=Scratch area at 024 h−scratch area at  h/scratch area at 0 h×100%.

For cell apoptosis, the Annexin V‐FITC apoptosis assay kit (Beyotime) was utilized. Briefly, MRC‐5 cells with different treatments were harvested and washed with pre‐chilled PBS. After centrifugation at 613 × *g* for 10 min at 4°C, the cells were resuspended with 500 μL of Annexin V‐FITC binding buffer, followed by the addition of 5 μL of Annexin V‐FITC and 5 μL of propidium iodide (PI). After thorough mixing, the cells were incubated in the dark for 30 min, and the cell apoptotic rate in each group was detected using a flow cytometer. The total apoptosis rate was calculated as the sum of early apoptotic cells (Annexin V‐FITC positive, PI negative, Q3 quadrant) and late apoptotic/secondary necrotic cells (Annexin V‐FITC positive, PI positive, Q2 quadrant). The formula is calculated as:

Total apoptosis rate %=Q23 cell percentage+Q cell percentage×100.

For cell differentiation, IF was used to test the expression of filamentous actin (F‐actin) in the different cells. MRC‐5 cells with different treatments were collected, washed, fixed, and permeabilized. Subsequently, the cells were incubated with 100 nmol/L rhodamine phalloidin working solution (Solarbio) for 30 min at room temperature in the dark. Following three washes with PBS, the cells were counterstained with 200 μL of PBS containing 100 nmol/L 4′,6‐diamidino‐2‐phenylindole (DAPI) for 3 min. After an additional three washes with PBS, images were captured using a fluorescence inverted microscope (Nikon, Japan).

### 2.9. DNA Damage in Cells Using Comet Assay

The effects of H‐2‐104 on DNA damage in MRC‐5 cells were detected by the comet assay as previously reported [24]. In brief, MRC‐5 cells from each group were obtained, digested into single‐cell suspensions, and then fixed on slides. After solidification, the slides were immersed in cell lysis buffer (100 mmol/L Na_2_EDTA, 2.5 mol/L NaCl, and 10 mmol/L Tris; pH adjusted to 10 with 4 mol/L NaOH; 1% Triton X‐100% and 10% DMSO were added and mixed thoroughly before use) for lysis. After 4 h, the slides were washed with PBS and then immersed in alkaline electrophoresis solution for DNA unwinding for 25 min, followed by electrophoresis for 30 min (25 V, 300 mA). After electrophoresis, the slides were washed three times with Tris‐HCl buffer (pH 7.5) and stained with PI in the dark for 10 min. After washing three times with ultrapure water, images were observed and captured under a fluorescence microscope, and the Olive tail moment (OTM) was calculated using CometScore software (TriTek, Sumerduck, VA, USA).

### 2.10. Real‐Time Quantitative PCR (RT‐qPCR)

Total RNA was extracted from the MRC‐5 cells with different treatments using the TRIzol reagent (Thermo Fisher Scientific) based on the recommendations of the manufacturer. Then, the isolated total RNA was reverse transcribe into cDNA via PrimeScript RT Reagent Kit (Takara, Beijing, China). The sequences of all primers were synthesized by Sangon Biotech (Shanghai) Co., Ltd (Shanghai, China) and presented in Table 1. The RT‐qPCR reaction was initiated at 95°C for 30 s; 95°C for 5 s and 60°C for 30 s for a total of 40 cycles, and then 72°C for 10 min. β‐actin was served as a housekeeping RNA, and the relative mRNA expressions of related genes were calculated using the 2^−ΔΔCt^ method.

**Table 1:** The sequences of all primers.

Primers	Sequences (5′–3′)
Collagen I	F: ACTGGTACATCAGCCCAAAC R: GGAACCTTCGCTTCCATACTC
Fibronectin	F: GAAAGACCAGCAGAGGCATAA R: CACTCATCTCCAACGGCATAA
α‐SMA	F: GGCATCCACGAAACCACCTA R: AATGCCTGGGTACATGGTGG
ATR	F: CTGCTGGCTTGAGACCTATTC R: CTGCTGACTTTGGTAGCATACA
RAD51	F: CGGTGGCACTGTCTACAATAA R: TTCAACACAGACCACCAGAC
H2AX	F: TGAGTTTGCTGGAAGGGAAATGGG R: TTTGGCTTCACGGCTGGCTATG
β‐Actin	F: GAGGTATCCTGACCCTGAAGTA R: CACACGCAGCTCATTGTAGA

### 2.11. Statistical Analysis

Data are presented as the mean ± standard deviation (SD) from three independent experiments. The sample size of animal groups was determined according to published studies on similar animal models to ensure statistical reliability. All statistical analyses were conducted using SPSS 22.0 software, and GraphPad Prism 9.0 software was employed for graph plotting. Before statistical analysis, the Shapiro–Wilk test and Levene’s test were used to verify the normality and homogeneity of variance of all data, respectively. All data met the requirements for parametric tests. Differences between two groups were evaluated by Student’s *t*‐test, while comparisons among multiple groups were performed via one‐way analysis of variance (ANOVA) followed by least‐significant difference (LSD) post hoc pairwise comparisons. A *p*‐value < 0.05 was considered to indicate a statistically significant difference.

## 3. Results

### 3.1. H‐2‐104 Improved Lung Function and Lung Tissue Pathology of *E. granulosus*‐Infected PF Mice

**Figure 1:**
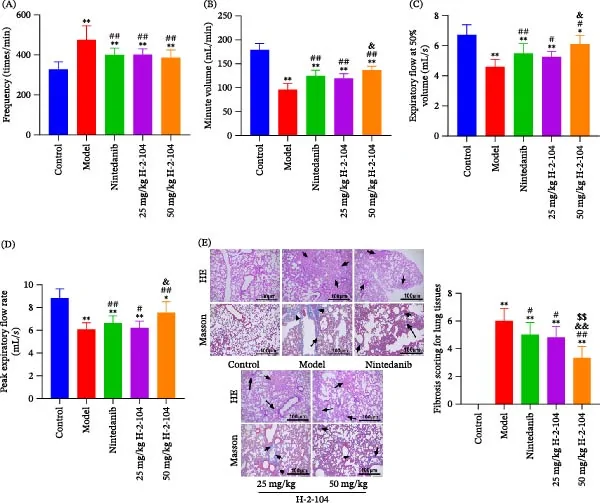
H‐2‐104 improved lung function and lung tissue pathology of *Echinococcus granulosus*‐induced pulmonary fibrosis (PF) mice. (A) The respiratory frequency of mice in different groups. (B) The minute ventilation (MV) of mice in different groups. (C) The expiratory flow at 50% ventilation (EF50) of mice in different groups. (D) The maximum expiratory flow rate (PEF) of mice in different groups. (E) Histopathologic examination and fibrosis scoring of lung tissues in mice using hematoxylin and eosin (HE) and Masson’s trichrome staining at a magnification of 100×. *N* = 10.  ^∗^ *p* < 0.05,  ^∗∗^ *p* < 0.01 vs. control; ^#^ *p* < 0.05, ^##^ *p* < 0.01 vs. model; ^&^ *p* < 0.05, ^&&^ *p* < 0.01 vs. Nintedanib; and ^$$^ *p* < 0.01 vs. 25 mg/kg H‐2‐104.

The progression of PF is closely associated with alterations in lung function, so we employed the EMMSLink WBP noninvasive whole‐body plethysmography system to evaluate respiratory function in mice. It was found that compared with the control group, the *E. granulosus*‐infected PF mice exhibited a significant increase in respiratory frequency (*p* < 0.01, Figure 1A), accompanied by marked decreases in multiple lung function parameters, including MV, EF50, and PEF (*p* < 0.01, Figure 1B–D). Notably, H‐2‐104 treatment significantly restored respiratory function in PF mice. Specifically, compared with the model group, the administration of 25 mg/kg H‐2‐104 significantly decreased the respiratory frequency (*p* < 0.05, Figure 1A), whereas increased the MV level (*p* < 0.05, Figure 1B). Furthermore, the administration of H‐2‐104 at 50 mg/kg significantly elevated the levels of MV, PEF, and EF50 (*p* < 0.05) while reducing the respiratory frequency (*p* < 0.05, Figure 1A–D) in comparison with the *E. granulosus*‐infected PF mice.

HE and Masson’s trichrome staining results revealed that the mice in the control group displayed intact alveolar structures with thin‐walled vacuolar alveolar walls, no thickening of alveolar septa, and no inflammatory cell infiltration in the lung interstitium (Figure 1E). In contrast, compared with the control group, the lung tissues of mice in the model group showed massive inflammatory cell infiltration in the alveolar cavities and lung interstitium. Additionally, the alveolar structures in the lesion areas were irregular, with alveolar cavity dilatation, atrophy, or disappearance, thickened alveolar septa, and partial fusion presenting as pulmonary consolidation; the alveoli in the surrounding areas were enlarged and deformed, with a significantly elevated fibrosis score relative to the control group (Figure 1E). After H‐2‐104 intervention, *E. granulosus* infection still induced the aforementioned pathological damage in lung tissues, but the severity was significantly alleviated, and the fibrosis score was markedly reduced (Figure 1E). Masson’s trichrome staining demonstrated that the model group had significantly more blue‐stained collagen fibers than the control group, while H‐2‐104 treatment remarkably reduced collagen fiber deposition (Figure 1E). Collectively, these results demonstrated that H‐2‐104 could ameliorate the impairment of lung function and pathological damage in mice with *E. granulosus*‐induced PF. Of note, 50 mg/kg H‐2‐104 exerted superior effects on improving lung function and reducing fibrotic lesions compared with nintedanib.

### 3.2. Effects of H‐2‐104 on HYP, Inflammatory Cytokines, and Fibrosis‐Related Protein Expression in PF Mice

**Figure 2:**
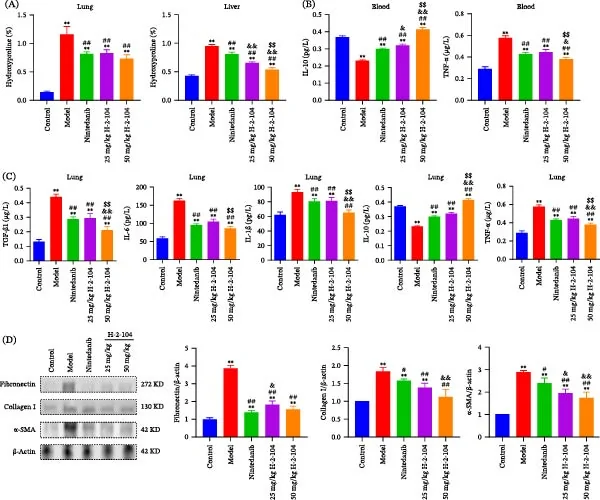
Effects of H‐2‐104 on hydroxyproline (HYP), inflammatory cytokines, and fibrosis‐related protein expression in PF mice. (A) The levels of HYP in lung and liver tissues of mice of different groups. (B) The levels of IL‐10 and TNF‐α in blood samples of mice in different groups. (C) The levels of TGF‐β1, IL‐6, IL‐1β, IL‐10, and TNF‐α in lung tissues of mice in different groups. (D) The protein expression levels of α‐SMA, collagen I, and fibronectin in mice of different groups using western blot. *N* = 3.  ^∗^ *p* < 0.05,  ^∗∗^ *p* < 0.01 vs. control; ^#^ *p* < 0.05, ^##^ *p* < 0.01 vs. model; ^&^ *p* < 0.05, ^&&^ *p* < 0.01 vs. Nintedanib; and ^$$^ *p* < 0.01 vs. 25 mg/kg H‐2‐104.

HYP is a specific marker for collagen metabolism, and its content directly reflects the degree of tissue fibrosis. We detected HYP levels in both lung and liver tissues. The pulmonary HYP contents in control and model groups were, respectively, 0.146 ± 0.009 µg/mg and 1.162 ± 0.108 µg/mg, which showed a significant increase of pulmonary HYP after *E. granulosus* infection compared to the control mice (*p* < 0.01, Figure 2A). However, the pulmonary HYP contents in the PF mice after administrated with nintedanib, 25 mg/kg H‐2‐104 and 50 mg/kg H‐2‐104 were 0.821 ± 0.026 µg/mg, 0.834 ± 0.046 µg/mg, and 0.733 ± 0.054 µg/mg, respectively, suggesting H‐2‐104, similar with nintedanib, evidently reduced the pulmonary HYP levels caused by *E. granulosus* (*p* < 0.01, Figure 2A). The trend of HYP levels in the liver tissues of different groups was consistent with that in lung tissues (Figure 2A). The elevated hepatic HYP content in model mice indicated obvious liver fibrosis, while H‐2‐104 treatment effectively reduced liver collagen deposition.

For circulating IL‐10 and TNF‐α levels in the blood samples, we found that circulating IL‐10 levels in the model group were significantly lower than in the control group, while nintedanib and H‐2‐104 treatments evidently increased its levels caused by the model group (Figure 2B). Moreover, 50 mg/kg H‐2‐104 exerted a superior effect relative to nintedanib and 25 mg/kg H‐2‐104 (Figure 2B). For circulating TNF‐α level, its tendency in the different groups was opposite to that of circulating IL‐10 levels (Figure 2B). We further calculated the TNF‐α/IL‐10 ratio based on the circulating cytokine data. The ratio was markedly elevated in model mice, indicating a shift toward a pro‐inflammatory state. Both nintedanib and H‐2‐104 reversed this imbalance, with H‐2‐104 showing a more potent regulatory effect.

In the lung tissues, the TGF‐β1 contents in the control, model, nintedanib, 25 mg/kg H‐2‐104, and 50 mg/kg H‐2‐104 groups were 0.132 ± 0.012 µg/L, 0.441 ± 0.016 µg/L, 0.287 ± 0.013 µg/L, 0.294 ± 0.027 µg/L, and 0.212 ± 0.019 µg/L, respectively (Figure 2C). The expression pattern of TGF‐β1 across groups was consistent with the changes in the HYP content (Figure 2C). TGF‐β1 is a multifunctional regulatory cytokine with predominant anti‐inflammatory properties, and it also acts as a central profibrotic mediator. Meanwhile, classical pro‐inflammatory cytokines, including TNF‐α (0.578 ± 0.015 µg/L vs. 0.293 ± 0.014 µg/L), IL‐6 (162.96 ± 4.55 pg/L vs. 58.70 ± 3.38 pg/L), and IL‐1β (93.74 ± 2.82 pg/L vs. 61.75 ± 3.48 pg/L), were markedly elevated in lung tissues of *E. granulosus*‐infected model mice compared with the control group (*p* < 0.01, Figure 2C). In contrast, compared with the PF model group, the nintedanib group and H‐2‐104 groups at different concentrations all significantly reduced the levels of TNF‐α, IL‐6, and IL‐1β in lung tissues (*p* < 0.01); as well as their levels in the 50 mg/kg H‐2‐104 group were even lower than those in the nintedanib group (*p* < 0.01, Figure 2C). Moreover, the trend of IL‐10 levels in lung tissues of different groups was opposite to that of TGF‐β1, IL‐6, IL‐1β, and TNF‐α levels (Figure 2C). These outcomes demonstrated that H‐2‐104 treatment could effectively reduce collagen production and deposition in lung tissues and exerted a significant therapeutic effect on inflammation during the progression of PF.

To further confirm the effects of H‐2‐104 on PF in vivo, western blot analysis was performed to detect the protein expression levels of α‐SMA, collagen I, and fibronectin. As shown in Figure 2D, the target protein bands of α‐SMA, collagen I, and fibronectin in the model group exhibited significantly higher intensity and abundance compared with the control group (*p* < 0.01), indicating elevated protein expression in the model group. Notably, compared with the model group, H‐2‐104 treatment significantly downregulated their protein expression induced by *E. granulosus* (*p* < 0.01), as well as 50 mg/kg H‐2‐104 showed better effects. These findings implied that H‐2‐104 may alleviate the progression of PF by downregulating the expression of various profibrotic factors (α‐SMA, collagen I, and fibronectin). Consistent with the pathological results, H‐2‐104 especially the 50 mg/kg group showed stronger efficacy in reducing collagen deposition and inflammatory mediator levels than nintedanib.

### 3.3. Preliminary Study on the Safety of H‐2‐104

**Figure 3:**
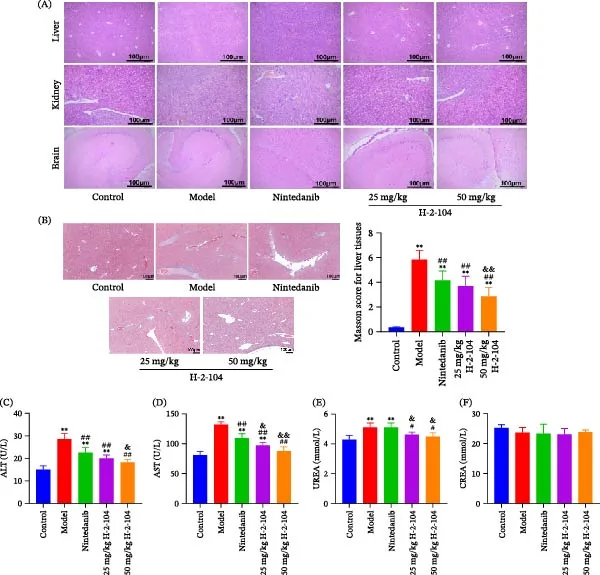
Preliminary study on the safety of H‐2‐104. (A) Histopathologic examination of the liver, kidney, and brain tissues in mice using HE staining at a magnification of 100×. *N* = 10. (B) Masson staining and score of the liver tissues in mice at a magnification of 100×. *N* = 10. (C) The levels of alanine aminotransferase (ALT) in mice of different groups. *N* = 3. (D) The levels of aspartate aminotransferase (AST) in mice of different groups. *N* = 3. (E) The levels of urea (UREA) in mice of different groups. *N* = 3. (F) The levels of creatinine (CREA) in mice of different groups. *N* = 3.  ^∗^ *p* < 0.05,  ^∗∗^ *p* < 0.01 vs. control; ^#^ *p* < 0.05, ^##^ *p* < 0.01 vs. model; and ^&^ *p* < 0.05, ^&&^ *p* < 0.01 vs. Nintedanib.

To preliminarily investigate the safety profile of H‐2‐104, HE staining combined with Masson staining was performed on the liver, kidney, and brain tissues of mice with PF, and blood biochemical indices were detected. In liver tissues, the model group exhibited massive inflammatory cell infiltration in the hepatic portal areas, along with dilation and congestion of portal areas, central veins, and hepatic sinusoids, with a higher Masson score in the model group. The nintedanib group showed aggravated hepatic inflammation and congestion, while H‐2‐104 treatment reduced the severity of hepatic inflammatory reactions and gradually alleviated the dilation and congestion of portal areas, central veins, and hepatic sinusoids, with lower Masson scores (Figure 3A,B). For kidney tissues, compared to the control group, the model mice presented with an abnormal renal structure, marked hyperemia in glomeruli and renal interstitium, and massive infiltration of pathological inflammatory cells. In contrast to the control mice, the nintedanib‐treated PF mice showed swelling of renal tubular epithelial cells, hyperemia and inflammatory cell infiltration in the renal interstitium, as well as partial renal tubular necrosis. However, the mice in the H‐2‐104 treatment groups exhibited no significant pathological changes in glomeruli or renal tubules, with a gradual alleviation of interstitial hyperemia and hemorrhage (Figure 3A). In addition, H‐2‐104 treatment also had no significant impact on the brain tissues of *E. granulosus*‐infected PF model mice (Figure 3A).

Blood biochemical analysis showed that the levels of ALT and AST were significantly elevated in the model group compared with the control group (*p* < 0.01), whereas they were markedly reduced in the PF mice‐treated with nintedanib, 25, and 50 mg/kg H‐2‐104 (*p* < 0.01), and 50 mg/kg H‐2‐104 had the most effect (Figure 3C,D). For UREA, its level was significantly increased in the nintedanib group relative to the control and model groups (*p* < 0.05), while no significant difference in its level was observed among the control, model, 25, and 50 mg/kg H‐2‐104 groups (*p* > 0.05, Figure 3E). Furthermore, there were no obvious alterations in CREA levels among the control, model, nintedanib, 25, and 50 mg/kg H‐2‐104 groups (*p* > 0.05, Figure 3F). Collectively, these findings suggested that H‐2‐104 could exert a certain protective effect on liver and kidney tissues while improving the pathological process of PF, and no significant toxicity to the brain tissue was observed, preliminarily confirming its safety at therapeutic doses. In contrast to nintedanib, which caused obvious hepatic and renal adverse reactions, H‐2‐104 displayed a better safety profile.

### 3.4. H‐2‐104 Inhibited the Viability of TGF‐β1‐Induced MRC‐5 Cells

**Figure 4:**
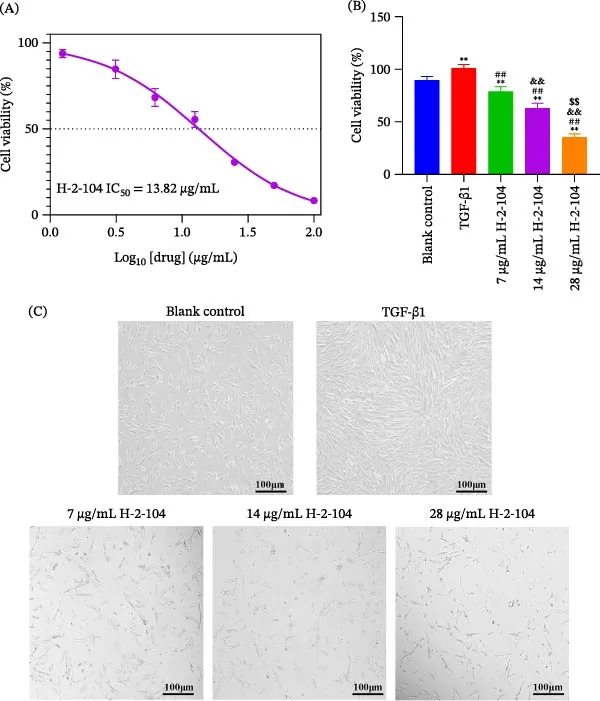
H‐2‐104 inhibited the viability of TGF‐β1‐induced MRC‐5 cells using cell counting kit‐8 (CCK‐8). (A) Inhibition of TGF‐β1‐stimulated MRC‐5 cell viability by different concentrations of H‐2‐104. (B) Based on the IC_50_ value, three concentrations (7, 14, and 28 µg/mL) of H‐2‐104 were selected for a 24‐h intervention to verify the cell viability. (C) The representative images of cell morphology after TGF‐β1 stimulation and different concentrations of H‐2‐104 treatments. *N* = 3.  ^∗∗^ *p* < 0.01 vs. control; ^##^ *p* < 0.01 vs. model; ^&&^ *p* < 0.01 vs. 7 µg/mL H‐2‐104; and ^$$^ *p* < 0.01 vs. 14 µg/mL H‐2‐104.

The CCK‐8 assay was utilized to investigate the roles of H‐2‐104 at different concentrations on the growth of TGF‐β1‐induced MRC‐5 cells. The results demonstrated that compared with the blank control group, the TGF‐β1 group significantly promoted the viability of MRC‐5 cells (*p* < 0.01). In contrast, when compared with the TGF‐β1‐induced MRC‐5 cells, the cell viability in each H‐2‐104 treatment group gradually decreased with the increase of H‐2‐104 concentrations. These indicated that H‐2‐104 could inhibit the viability of TGF‐β1‐induced MRC‐5 cells in a concentration‐dependent manner, with an IC_50_ value of 13.49 µg/mL (Figure 4A).

Based on the IC_50_ value, three concentrations (7, 14, and 28 µg/mL) of H‐2‐104 were selected for a 24‐h intervention to verify cell viability. Consistent with the previous findings, TGF‐β1 stimulation effectively promoted the viability of MRC‐5 cells, while all the three concentrations of H‐2‐104 exerted a significant inhibitory effect on the viability of TGF‐β1‐induced MRC‐5 cells, with 28 µg/mL H‐2‐104 showing the best inhibitory effects (*p* < 0.01, Figure 4B). Additionally, the cell morphology after TGF‐β1 stimulation and different concentrations of H‐2‐104 treatments was displayed in Figure 4C.

### 3.5. H‐2‐104 Inhibited Migration and Differentiation While Promoted Apoptosis of TGF‐β1‐Induced MRC‐5 Cells

**Figure 5:**
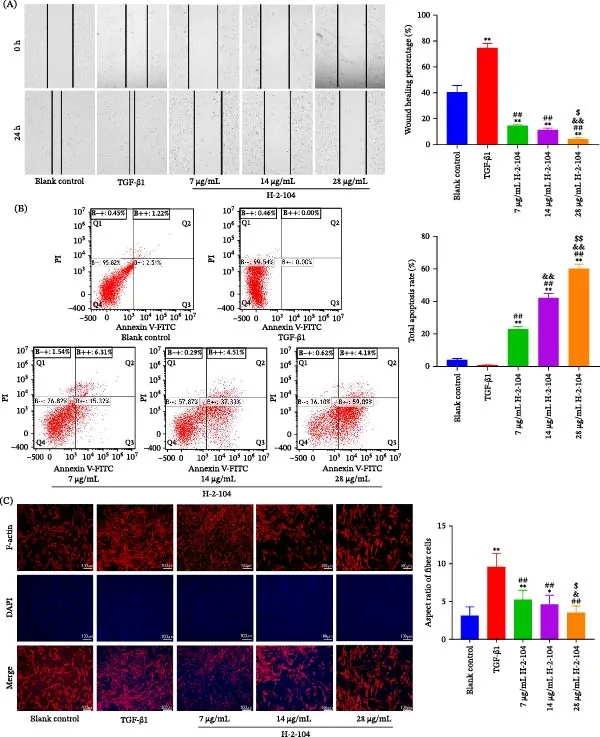
H‐2‐104 inhibited migration and differentiation while promoted apoptosis of TGF‐β1‐induced MRC‐5 cells. (A) Effects of H‐2‐104 on the migration of TGF‐β1‐induced MRC‐5 cells using scratch assay. (B) Effects of H‐2‐104 on the apoptosis of TGF‐β1‐induced MRC‐5 cells by flow cytometry. Flow cytometry scatter plots are fully labeled with *X*‐axis (Annexin V‐FITC) and *Y*‐axis (PI); total apoptosis rate was calculated by summing Q2 and Q3 quadrant cell proportions. (C) Effects of H‐2‐104 on the formation of cytoskeletal filaments (F‐actin) in TGF‐β1‐induced MRC‐5 cells by immunofluorescence (IF). *N* = 3.  ^∗^ *p* < 0.05,  ^∗∗^ *p* < 0.01 vs. control; ^#^ *p* < 0.05, ^##^ *p* < 0.01 vs. model; ^&^ *p* < 0.05, ^&&^ *p* < 0.01 vs. 7 µg/mL H‐2‐104; and ^$^ *p* < 0.05, ^$$^ *p* < 0.01 vs. 14 µg/mL H‐2‐104.

The scratch assay was performed to verify the inhibitory effect of H‐2‐104 on the migration of TGF‐β1‐induced MRC‐5 cells. The results showed that TGF‐β1 induction significantly promoted cell migration compared with the blank control group (*p* < 0.01), as evidenced by reduced scratch width and a 34.29% increase in the migration rate of MRC‐5 cells relative to the control group (Figure 5A). After H‐2‐104 intervention, cell migration was evidently suppressed compared with the TGF‐β1 group (*P* < 0.05), accompanied by widened scratch width (Figure 5A). Specifically, the cell migration rates were reduced by 59.88%, 63.49%, and 70.42%, respectively, in the 7, 14, and 28 μg/mL H‐2‐104‐treated groups compared with the TGF‐β1 group (Figure 5A). Then, flow cytometry analysis demonstrated that H‐2‐104 significantly induced dose‐dependent apoptosis in MRC‐5 cells. Compared with the apoptosis rate of the control group (1.42%), treatment with 7, 14, and 28 μg/mL H‐2‐104 increased the cell apoptosis rate to 24.06%, 42.71%, and 60.81%, respectively (*p* < 0.01, Figure 5B). In addition, the formation of cytoskeletal filaments is a critical phenotypic marker for the trans‐differentiation of lung fibroblasts into myofibroblasts. IF staining of cytoskeletal filaments in lung fibroblasts displayed that compared with the blank control group, TGF‐β1‐induced MRC‐5 cells exhibited a significant increase in F‐actin expression. In contrast, H‐2‐104 at concentrations of 7, 14, and 28 μg/mL remarkably downregulated the expression of F‐actin (red fluorescence), with 28 μg/mL H‐2‐104 having the best action (Figure 5C). Taken together, these findings demonstrated that H‐2‐104 intervention could effectively inhibit the migration while promoting the apoptosis of TGF‐β1‐induced MRC‐5 cells, as well as suppress the phenotypic transition of lung fibroblasts to myofibroblasts.

### 3.6. Effects of H‐2‐104 on DNA Damage and Expression of Fibrosis‐ and DNA Damage‐Related Genes In Vitro

**Figure 6:**
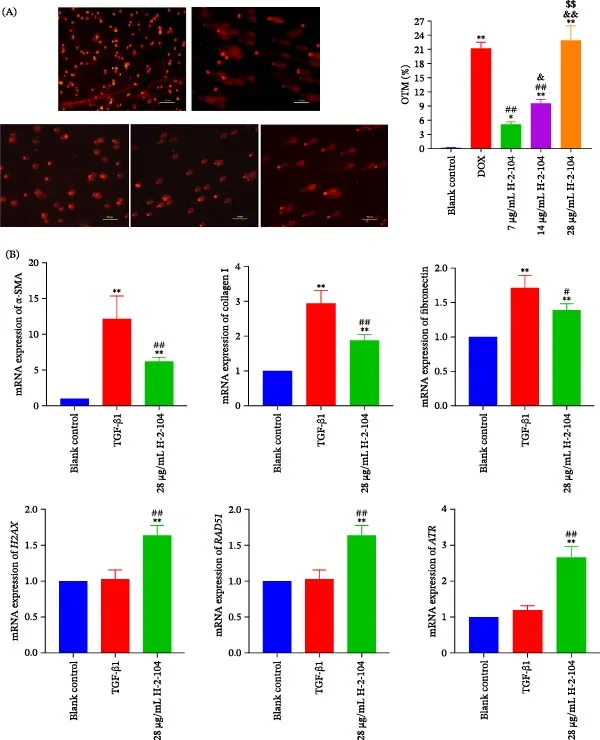
Effects of H‐2‐104 on DNA damage and expression of fibrosis‐ and DNA damage‐related genes in vitro. (A) The effects of H‐2‐104 on DNA damage (Olive tail moment [OTM] values) in TGF‐β1‐induced MRC‐5 cells detected by comet assay. *N* = 4. (B) The mRNA expression of fibrosis‐ (α‐SMA, collagen I, and fibronectin) and DNA damage‐related (*H2AX*, *ATR*, and *RAD51*) genes in MRC‐5 cells with different treatments using RT‐qPCR. *N* = 3.  ^∗^ *p* < 0.05,  ^∗∗^ *p* < 0.01 vs. control; ^#^ *p* < 0.05, ^##^ *p* < 0.01 vs. model; ^&^ *p* < 0.05, ^&&^ *p* < 0.01 vs. 7 µg/mL H‐2‐104; and ^$^ *p* < 0.05, ^$$^ *p* < 0.01 vs. 14 µg/mL H‐2‐104.

Given that H‐2‐104 exerted prominent antifibrotic and anti‐inflammatory effects in *E. granulosus*‐infected mice, we further explored its action mechanism at the cellular level. Changes in the cell membrane and cytoplasm occur during the early and middle stages of apoptosis, while DNA damage and fragmentation in the nucleus are characteristic of the late stage. Thus, DNA fragmentation can be detected to reflect the apoptotic status of cells. The alkaline comet assay results showed that TGF‐β1‐induced MRC‐5 cells exhibited DNA damage following treatment with doxorubicin (DOX). Compared with the blank control group, the DOX group had a significantly increased level of DNA damage (*p* < 0.01, Figure 6A). Notably, H‐2‐104 significantly increased cellular DNA damage in MRC‐5 cells in a dose‐dependent manner, as evidenced by the elevation of the OTM from 0.21% in the control group to 4.97%, 10.11%, and 23.48% in the 7, 14, and 28 μg/mL H‐2‐104‐treated groups (*p* < 0.01, Figure 6A). Furthermore, the OTM value of MRC‐5 cells treated with 28 μg/mL H‐2‐104 was comparable to that of the DOX group (Figure 6A). These data indicated that H‐2‐104 treatment could promote DNA damage in TGF‐β1‐stimulated MRC‐5 cells, thereby exerting a significant inhibitory effect on cell survival via inducing apoptosis.

Finally, RT‐qPCR was used to determine the mRNA expression of fibrosis‐ (α‐SMA, collagen I, and fibronectin) and DNA damage‐related (*H2AX*, *ATR*, and *RAD51*) genes in MRC‐5 cells with different treatments. We observed that the control MRC‐5 cells exhibited minimal expression of α‐SMA, collagen I, and fibronectin, while following TGF‐β1 stimulation, the mRNA expression levels of α‐SMA, collagen I, and fibronectin were significantly upregulated compared with the blank control cells (*p* < 0.01, Figure 6B). However, H‐2‐104 intervention remarkably reduced the mRNA expression levels of these profibrotic genes (α‐SMA, collagen I, and fibronectin) relative to the TGF‐β1 group (*p* < 0.01, Figure 6B). For DNA damage‐related genes (*H2AX*, *ATR*, and *RAD51*), no significant differences in their mRNA expression were found among the blank control and TGF‐β1 groups (*p* > 0.05), however, H‐2‐104 significantly upregulated their mRNA expression relative to the blank control and TGF‐β1 groups (*p* < 0.01, Figure 6B). These findings suggested that H‐2‐104 could attenuate TGF‐β1‐induced fibroblast‐to‐myofibroblast transdifferentiation. The altered expression of DNA damage‐related genes and elevated DNA damage were observed alongside the antifibrotic phenotypes in H‐2‐104‐treated cells, implying that the DNA damage response may contribute to its cellular effects.

## 4. Discussion

PE‐induced PF is a devastating complication of CE, representing a complex and dynamic process involving multiple cell types throughout the pathological fibrotic cascade [25–27]. A prominent feature of this disease is the abnormal activation of lung fibroblasts into pathological myofibroblasts, which leads to excessive ECM production and the formation of progressive fibrotic scars within the lung parenchyma, thereby severely impairing lung function and threatening patient survival [28–30]. Despite advances in antifibrotic therapy, the approved antifibrotic agents nintedanib and pirfenidone cannot reverse fibrosis and are associated with organ toxicity, which hinders their long‐term administration [31, 32]. Relative to approved first‐line antifibrotic agents, H‐2‐104 exerted stronger pharmacological effects and presented negligible organ toxicity in our animal experiments. Natural product derivatives have emerged as promising candidates for antifibrotic drug development due to their diverse biological activities and favorable safety profiles. In this study, we systematically demonstrated that HM derivative H‐2‐104 could alleviates *E. granulosus*‐induced PF in mice and inhibit TGF‐β1‐mediated fibroblast activation in vitro, providing novel insights into the treatment of this refractory disease.

The pathological progression of PF is closely linked to impaired lung function, which serves as a key clinical endpoint for evaluating therapeutic efficacy [33, 34]. Our in vivo results demonstrated that *E. granulosus* infection significantly increased respiratory frequency and decreased MV, EF50, and PEF in mice, consistent with the typical functional impairment observed in PF [35, 36]. H‐2‐104 treatment dose‐dependently restored these lung function parameters, accompanied by improved lung tissue pathology, characterized by reduced inflammatory cell infiltration, alveolar structure disruption, and collagen fiber deposition. These findings are consistent with previous studies showing that effective antifibrotic agents ameliorate both functional and structural abnormalities in PF models [37, 38]. Notably, H‐2‐104 at 50 mg/kg exhibited comparable or even superior efficacy to nintedanib in improving lung function, inhibiting inflammation and reducing collagen accumulation across multiple detection indicators. Combined with safety evaluation data, H‐2‐104 showed distinct advantages in protecting liver and kidney tissues and avoiding adverse reactions. All inter‐group statistical differences demonstrate the comprehensive superiority of H‐2‐104 and highlight its great potential as a novel therapeutic alternative for clinical application.

Fibroblast activation and subsequent myofibroblast differentiation are central to the pathogenesis of PF, as myofibroblasts are the primary producers of ECM components such as collagen I and fibronectin [33, 39]. A prominent feature of myofibroblasts is the de novo expression of α‐SMA in stress fibers, which serves as the molecular basis for their high contractile activity [40]. During normal wound healing, myofibroblasts arise through multiple pathways, predominantly via fibroblast differentiation, and are regulated by factors including TGF‐β1, fibronectin, and tissue stiffness [41]. In the setting of parasitic infection, sustained inflammatory stimulation triggers a compensatory increase in TGF‐β1, which serves as a physiological regulatory feedback mechanism to limit excessive inflammatory responses and protect tissues from further damage. TGF‐β1 is a multifunctional regulatory cytokine with major anti‐inflammatory activities. It also serves as the core profibrotic factor that drives myofibroblast formation, directly promoting ECM synthesis and α‐SMA expression [42]. Through activation of Smad‐dependent signaling pathways, TGF‐β1 stimulates fibroblasts to transdifferentiate into myofibroblasts while upregulating ECM component deposition, leading to tissue stiffening that further exacerbates the fibrotic cascade [43]. Excessive collagen deposition is a hallmark of fibrotic diseases, and HYP, an amino acid uniquely abundant in collagen (accounting for ~13% of total amino acids in collagen), makes its total content a reliable indicator for assessing the severity of fibrosis [44]. In addition, the correlation between fibrosis and inflammation has been confirmed and supported by morphological evidence [45, 46]. Of note, distinct from classical pro‐inflammatory cytokines, such as TNF‐α and IL‐1β, TGF‐β1 mainly exerts immunomodulatory and anti‐inflammatory effects, while its potent profibrotic activity plays a decisive role in the progression of PF. In the present study, *E. granulosus* infection significantly increased the levels of HYP, TGF‐β1, and pro‐inflammatory cytokines (TNF‐α, IL‐6, and IL‐1β) in lung tissues and remarkably upregulated the expression of profibrotic related proteins (α‐SMA, collagen I, and fibronectin). However, H‐2‐104 treatment significantly reduced the levels of these pro‐inflammatory mediators and concurrently downregulated the expression of profibrotic proteins. Li et al. [47] reported that salidroside could down‐regulate the expression of α‐SMA, vimentin, TGF‐β1, snail and slug, while reducing the release of inflammatory cytokines (IL‐1β, IL‐6, and TNF‐α), thereby inhibiting the TLR4/NF‐κB and MAPK signaling pathways to alleviate renal interstitial fibrosis. Taken together, we speculate that H‐2‐104 may suppress *E. granulosus*‐induced PF via inhibiting the TGF‐β1‐mediated profibrotic cascade. Unlike previous research that focused on HM in idiopathic fibrosis [18], our work verifies this pharmacological effect in a unique parasitic infection‐induced fibrotic model and further extends the relevant mechanistic research to the field of echinococcosis‐associated PF.

To further elucidate the cellular mechanisms underlying the antifibrotic effect of H‐2‐104, we used TGF‐β1‐activated MRC‐5 human lung fibroblasts, a well‐established in vitro model of fibroblast activation [48, 49]. Functional assays showed that H‐2‐104 dose‐dependently suppressed fibroblast proliferation and migration (IC_50_ = 13.49 µg/mL). Flow cytometry analysis revealed that H‐2‐104 promoted fibroblast apoptosis, which is consistent with previous findings that inducing apoptotic cell death of activated fibroblasts is a critical antifibrotic strategy [50, 51]. IF staining further confirmed that H‐2‐104 reduced the formation of F‐actin, a key phenotypic feature of fibroblast‐to‐myofibroblast trans‐differentiation [52, 53]. Collectively, these in vitro data suggest that H‐2‐104 may target multiple cellular processes essential for fibrosis progression, incorporating fibroblast activation, proliferation, migration, and differentiation.

DNA damage and repair mechanisms have recently emerged as key regulators of fibroblast function and fibrosis development [54]. A major innovation of the present study is that we first revealed the correlation between the DNA damage response and fibroblast inactivation in *E. granulosus*‐induced PF. This mechanistic link has long been an underexplored research gap in this specific fibrotic subtype. Consistent with the in vivo antifibrotic and anti‐inflammatory effects of H‐2‐104 in *E. granulosus*‐induced PF mice, our in vitro cellular experiments further revealed the phenotypic changes in activated fibroblasts. Our comet assay results showed that H‐2‐104 increased DNA damage levels in TGF‐β1‐activated MRC‐5 cells in a dose‐dependent manner, as evidenced by increased OTM values comparable to those induced by DOX, a known DNA‐damaging agent. RT‐qPCR analysis further revealed that H‐2‐104 upregulated the mRNA expression of DNA damage‐related genes (*H2AX*, *ATR*, and *RAD51*), which are critical for sensing and repairing DNA lesions. H2AX, a crucial participant in the DNA damage response [55], is reported to be activated by Nlgn173 protein, thereby mediating doxorubicin‐stimulated cardiac remodeling and fibrosis [56]. A previous study of Zhang et al. [57] demonstrated that YTHDC1 could activate ATR in a non‐m6A manner and promote DNA damage repair, thus delaying cellular aging and PF. High expression of RAD51 promotes DNA damage repair and survival in KRAS mutant lung cancer cells [58]. It has been shown that RAD51 and BRCA2 signaling dependent on FoxM1 can protect idiopathic PF fibroblasts from radiation‐induced cell death through the DNA damage repair pathway [59]. In our cellular model, H‐2‐104 downregulated the mRNA expression of profibrotic genes (α‐SMA, collagen I, and fibronectin) while upregulating DNA damage‐related genes. This observed correlation implies that the altered DNA damage response may be associated with fibroblast inactivation. However, the interplay between DNA damage repair mechanisms and fibroblast activation in the context of *E. granulosus*‐induced PF has rarely been investigated. These reports, together with our findings, lead us to speculate that changes in the DNA damage response in fibroblasts may be one of the multiple pathways participating in the antifibrotic action of H‐2‐104. This finding not only enriches the mechanistic network of parasitic fibrosis but also provides a brand‐new target direction for the development of antifibrotic drugs.

Safety is a critical consideration for antifibrotic drug development, as long‐term administration is often required for chronic fibrotic diseases [60]. HM, the parent compound of H‐2‐104, has limited clinical application due to its significant neurotoxicity. However, our safety assessment showed that H‐2‐104 treatment did not cause obvious pathological damage to the liver, kidney, or brain tissues of mice. Moreover, H‐2‐104 significantly reduced the elevated levels of ALT and AST in *E. granulosus*‐infected mice, which implies a protective effect on hepatic function. In contrast, nintedanib treatment increased hepatic inflammatory responses and renal tubular damage, consistent with its known adverse effects on the gastrointestinal and renal systems [61, 62]. These findings indicate that H‐2‐104 may have a more favorable safety profile compared that of existing therapies. While current clinical antifibrotic drugs inevitably cause organ damage and gastrointestinal side effects during long‐term administration, H‐2‐104 showed no neurotoxicity (the major defect of its parent compound HM) and exerted protective effects on liver and kidney tissues, which makes it a safer alternative for the long‐term intervention of chronic fibrotic diseases. Further long‐term and dose‐escalation studies are still warranted.

However, several limitations of this study should be acknowledged. First, although we confirmed that H‐2‐104 suppresses fibroblast activation and alters the cellular DNA damage status, future studies should investigate whether H‐2‐104 interacts with key signaling molecules in the TGF‐β1/Smad pathway or DNA damage repair machinery. Second, our in vitro experiments were performed using only the MRC‐5 human lung fibroblast cell line, which may limit the general applicability of the cellular results. In addition, the in vivo safety assessment was carried out within a 21‐day short‐term treatment period; long‐term toxicological profiles require further investigation. Third, clinical translation will require validation in large animal models and human clinical trials. Fourth, we mainly focused on fibroblast‐mediated mechanisms, but the roles of other cell types, such as macrophages and epithelial cells, in the antifibrotic effect of H‐2‐104 warrant further exploration. Notably, markers for macrophage infiltration and polarization were not examined in this study. Given the altered TNF‐α/IL‐10 ratio, macrophage‐associated inflammation likely participates in disease progression, and we will explore this topic in future work.

In conclusion, our study demonstrates for the first time that the HM derivative H‐2‐104 attenuates *E. granulosus*‐induced PF in mice. Its antifibrotic actions are associated with fibroblast regulation, inflammatory modulation, and altered DNA damage status. Compared with the first‐line clinical drug nintedanib, H‐2‐104 achieves comparable or better antifibrotic efficacy and possesses superior biosafety, completely overcoming the neurotoxicity of its parent compound harmine. Furthermore, three core innovations of this work include the use of a clinically relevant *E. granulosus*‐induced PF model, confirmation of H‐2‐104’s advantages in efficacy and safety, and discovery of a novel link between DNA damage response and fibroblast function. These findings identify H‐2‐104 as a promising candidate for treating *E. granulosus*‐induced PF and provide a theoretical basis for the further development of HM derivatives as antifibrotic agents.

## Author Contributions

**Yuehong Gong:** conceptualization, methodology, writing – original draft preparation. **Yating Cui:** methodology, investigation, formal analysis. **Miechi Pan:** investigation, data curation, software. **Yisikandier Abudusaimaiti:** methodology, investigation, software. **Guohua Tang and Haibo Zhang**: methodology, formal analysis. **Hang Ren and Lijie Fu**: investigation, formal analysis. **Jianhua Yang:** conceptualization, supervision, writing – review and editing. **Junping Hu:** conceptualization, supervision, funding acquisition, writing – review and editing.

## Funding

This study was supported by the “Tianshan Talents” High‐Level Medical and Health Personnel Training Plan of the Health Commission of Xinjiang Uygur Autonomous Region (Grants TSYC202301B095 and TSYC202401B156), and the Xinjiang Uyghur Autonomous Region College Students Innovation Training Program Project (Grant S202510760018).

## Disclosure

All authors have reviewed and approved the submitted version of the manuscript. The funding body played no role in the design of the study and collection, analysis, and interpretation of data, and in writing the manuscript.

## Ethics Statement

This study was reviewed and approved by the Ethics Committee of the Animal Center of Xinjiang Medical University, and all procedures were performed in strict accordance with ethical standards (Approval Number: IACUC‐20230321‐54).

## Consent

The authors have nothing to report.

## Conflicts of Interest

The authors declare no conflicts of interest.

## Data Availability

The dataset used and/or analyzed during the current study are available from the corresponding author upon a reasonable request.
